# 

**Published:** 1896-06-06

**Authors:** 


CADBURY'S

COCOA

"The standard of highest purity at present attainable."—*Lancet.*

CADBURY'S

COCOA

NO ALKALIES USED.

### THE HOSPITAL

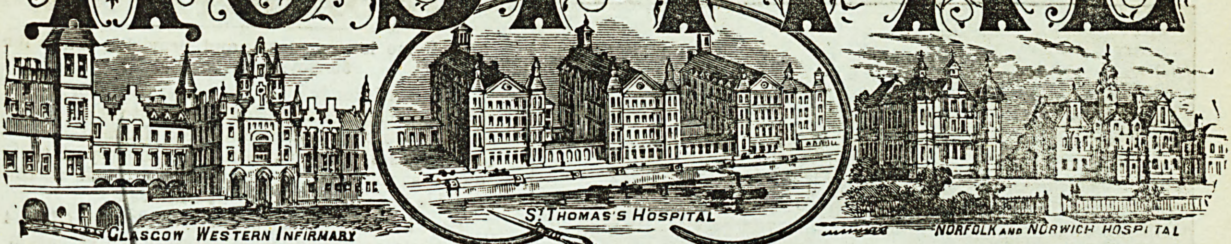

No. 506. Vol. XX.

SATURDAY JUNE 6TH, 1896.

WITH EXTRA NURSING SUPPLEMENT.
[Registered at the General Post Office as a Newspaper for transmission in the United Kingdom and Abroad.]

PRICE TWOPENCE
Per Annum, 10s. 6d., post free.
Monthly Parts, 6d.; post free, 8d.
Ditto per Annum, 8s. 6d., post free.

